# Challenges and Opportunities for Dental Education from COVID-19

**DOI:** 10.3390/dj10100188

**Published:** 2022-10-06

**Authors:** Bolei Li, Lei Cheng, Haohao Wang

**Affiliations:** 1 State Key Laboratory of Oral Diseases, West China School of Stomatology, National Clinical Research Center for Oral Diseases, Sichuan University, Chengdu 610041, China; 2 Department of Operative Dentistry and Endodontics, West China School of Stomatology, Sichuan University, Chengdu 610041, China

**Keywords:** COVID-19, dental education, distance education, teledentistry, dental emergency

## Abstract

With the ongoing COVID-19 pandemic, dental education has been profoundly affected by this crisis. First of all, COVID-19 brought physical and psychological health problems to dental students and educators. In addition, both non-clinical teaching and clinical-based training experienced challenges, ranging from fully online educational content to limited dental training, students’ research was delayed in achieving project milestones and there was hesitancy in respect of the COVID-19 vaccine. On the other hand, the COVID-19 pandemic has increased the demand for teledentistry and dental emergency treatment, and brought awareness of the advantages and high-speed development of distance education. This review aims to present these challenges and opportunities for dental education, and suggest how dental institutions should prepare for the future demand for dental education.

## 1. Introduction

Coronavirus disease (COVID-19) is an infectious disease caused by the SARS-CoV-2 virus. The modes of transmission for SARS-CoV-2 mainly include contact, droplet, airborne, and fomite. As of 1 September 2022, the overall number of confirmed cases and mortalities are 603,525,541 and 6,496,790, respectively, according to data from the World Health Organization (WHO), and more than 10 countries have accumulated at least 10 million confirmed cases, in particular the United States with 90 million cases. Currently, the Omicron variant is spreading all over the world. It spreads more easily than the original virus that causes COVID-19 and the Delta variant, but causes less severe disease.

**Figure 1:**
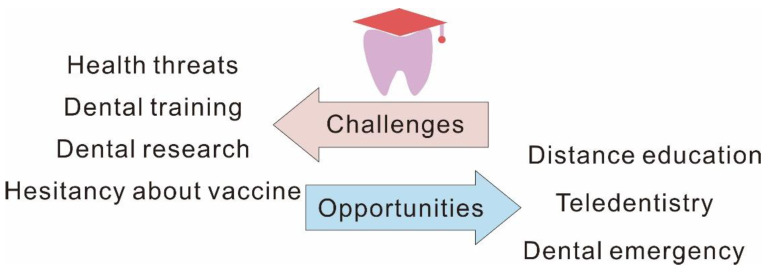
Challenges and opportunities for dental education from COVID-19.

Dental education involves the teaching and learning of the future generations of dentists to prevent, diagnose and treat oral diseases and meet the dental needs and demands of individual patients and the public. Dental schools are considered competitive and demanding learning communities. Dental education usually consists of two primary components over several years, non-clinical teaching and clinical-based training. In clinical-based training, students participate in clinical work, facing patients, instead of books or machines. The clinical-based training is indispensable in changing dental students into dentists. It demands of the students both intellectual and technical skills, including a sensitivity to patients’ needs as well as technical ability and, most critically, risk consideration. The ongoing COVID-19 pandemic has resulted in devastating consequences, not only in terms of economic, social, and health problems, but it has also impacted dental education [1]. On the one hand, the COVID-19 pandemic raised challenges for participants in dental education. In high exposure risk conditions, dental students suffered both physical health crises and psychological stress. Furthermore, dental students’ hesitancy in respect of the COVID-19 vaccine could increase the challenges. On the other hand, it raised challenges to the course of dental education. In response to the government’s epidemic prevention and control policies, many dental schools and professors worked from home and provided remote courses and examinations. Both non-clinical teaching and clinical-based training experienced challenges, ranging from fully online educational content to limited dental training. Much dental research has been delayed or even halted. However, the devastating consequences to traditional dental healthcare caused by the COVID-19 pandemic brought a new awareness of teledentistry and dental emergency to people, and provided a magnifying glass to view the advantages of distance education [1]. Considering the far-reaching impact of the pandemic on society, we wish to summarize the challenges and opportunities surrounding COVID-19, to prepare for the future demand for dental education (Figure 1).

## 2. Materials and Methods

An electronic search was conducted using PubMed, Google Scholar, and Web of Science, to identify relevant articles published between January 2020 and May 2022. Keywords such as ‘dental student’, ‘dental education’, ‘health’, ‘psychological’, ‘mental’, ‘training’, ‘teaching’, ‘dental research’, ‘practice’, ‘COVID-19 vaccine’, ‘distance education’, ‘teledentistry’, ‘e-dentistry’, ‘dental emergency’, ‘COVID-19′, and ‘pandemic’ were applied for each database in conjunction with the use of Boolean operators ‘AND’ and ‘OR’. Studies focusing only on the general population or specific samples that were not dental students were excluded. In addition, some studies beyond the research were also included in the review to discuss the viewpoints.

## 3. Results

During the initial search, a total of 482 articles were retrieved, A total of 107 duplicate articles were deleted, followed by 317 articles being discarded after screening based on titles and abstracts. After reading the remaining 58 articles in full, we finally included 45 in our review according to exclusion reasons, including those that were non-dental related, and studies conducted before COVID-19.

### 3.1. Challenges in the Physical and Psychological Health of Dental Students and Educators

The practice of dentistry frequently involves the use of instruments such as dental turbines, micro-motor or rotary handpieces, ultrasonic scalers, and air-water syringes that create sprays containing droplets of water, saliva, blood, microorganisms, and other body fluids, particulates, and debris, all of which can contribute to the generation of aerosolized droplets and thus the transmission of SARS-CoV-2 [2,3]. Performing or being present for aerosol-generating procedures performed on known or suspected COVID-19 patients was defined as a very high-risk activity [4]. In dental procedures, ultrasonic scalers and high-speed handpieces are consistently considered high-risk aerosol-generating [2,5]. Therefore, in high exposure risk conditions, the physical health of dental students and educators suffers potential threats from COVID-19. A survey of Australasians revealed that high numbers of educators and students (90% and 93%, respectively) perceived their health to be at risk while working [6]. Dental students were significantly more worried than non-dental students about being infected with COVID-19. Fortunately, the cumulative COVID-19 infection prevalence rate among U.S. dentists was 2.6% as of November 2020, according to the American Dental Association (ADA). The prevalence in dentists was much lower than that of front-line healthcare workers (29.26%) [7], probably because most dental hospitals and dental clinics was closed, and only the emergency unit was open for patients.

COVID-19 not only brought physical health crises but also led to psychological stress, tension, anxiety, fear, and despair among affected populations. A meta-analysis showed a prevalence of anxiety of 35% and a prevalence of depression of 37% reported by dental students [8,9]. However, there was significant heterogeneity between studies, ranging from 19–76% for anxiety and 11–75% for depression [8,9]. The most significant difference in anxiety and depression was found between geographical regions. A lower prevalence of anxiety and depression was reported in Europe, while levels were usually higher in Asia [8,9]. Surprisingly, in Australia, COVID-19 increased the stress levels of 80% of dental educators and 87% of dental students [6]. Some studies found that female students reported significantly more anxiety [10,11,12]. In addition, single, junior students, and those who lived alone were more likely to experience psychological problems [11,12,13]. Besides contracting COVID-19, the stress of dental students mainly came from exams, losing manual dexterity skills, professional growth, and transmitting the disease to their family members and/or flat mates due to working in clinical practice. Sometimes, contracting COVID-19 was not their primary concern [6,10].

### 3.2. Challenges in Dental Non-Clinical Teaching and Clinical-Based Training

Due to lockdowns and guidelines from governments to reduce the transmission of COVID-19, dental teaching activities permitted in universities were very limited. Only emergency dental treatments or urgent, non-delayable dental treatments. with a preference for providing dental care for vulnerable patients, were permitted in the global world. Considering that students had to stay home, dental schools planned to change dental education programs [14,15,16,17].

Many dental schools and professors worked from home and provided remote courses and examinations [6,18,19]. Electronic platforms, video conferencing networks, and social media were being used for non-clinical teaching [6,20]. In a survey from the Association of Dental Education in Europe focusing on European management of the COVID-19 crisis between 25 March and 5 April 2020, 90% of dental schools used online pedagogical software tools, 72% used live or streamed videos, 48% provided links to further online materials, 65% participated in organizing virtual meetings and, less frequently, small-scale working [15]. However, most students suffered some difficulties with online courses. A multi-institutional survey including 4475 students from the US, Spain, Ireland, Chile, India, and Brazil between April 10 and July 5, 2020 showed that online courses went “smoothly with some troubles” for 51.8% of the respondents [21]. For example, 83% of students learning dental anatomy experienced a lack of proper hardware, high bandwidth, and strong internet connections, which were a potential barrier in online courses [22] (Table 1).

Without a doubt, the impacts of the COVID-19 crisis on clinical-based training are extreme. In the survey focusing on European dental schools, clinical work was mainly performed by 96% of senior staff. Thirty percent of postgraduate students took part in clinical work, while only 11% of undergraduate students were asked to help only in non-clinical activities [15]. At the University of Jordan, 87% of dental students thought their clinical training was negatively affected the most [23]. A survey of Australasians revealed that a predominance of educators (85%), and 44% of students considered the impacts of suspension on students’ clinical competence to be extreme [6]. However, facing the COVID-19 crisis, 90% of educators and 87% of students agreed that a temporary lockdown would help in the containment of the COVID-19 outbreak and minimize risks of infection [6].

As a result, schools were considering postponing the evaluation of required clinical competencies and planning to change their assessment schedule, particularly with regard to clinical hours, rather than reducing the clinical requirements. In Canada, requirements for successful graduation from dental school were changed [24]. In the mainland of China, standardized training examinations for dentists were postponed and students were required to pay for several more months of school to accumulate sufficient clinical experience before graduating. Something similar happened in the Hong Kong region of China [17]. Some examinations took place online in Hong Kong [17]. In Europe, dental schools postponed formative (46%) and summative assessments (42%) or organized examinations entirely online (50%) [15].

### 3.3. Challenges in Dental Research

For most dental students, dental research is a part of their curriculum. In response to the COVID-19 pandemic, academic institutions have scaled back research activities. Most of the institutions have taken measures to ensure laboratories’ safety by limiting the number of animals to only crucial and critical research activities and have instituted curbs to maintain only rare genetic lines of animals [16]. Some schools even shut down their lab for a period of time [16]. In addition, acquiring human tissue samples, like extracted teeth for dental research, is getting more difficult over time, because the lockdowns limited dental clinic activity [25]. Extra human resources, time, and cost were needed for laboratory research. Ongoing clinical studies and community trials have been fragmented due to the COVID-19 pandemic [25]. Lockdowns and guidelines from governments halted the clinical studies. Furthermore, lockdowns, travel disruptions, or even infection with COVID-19 led to some recruited participants being excluded from an ongoing trial. Therefore, many researchers, especially PhDs and other early-career researchers, were not able to complete their laboratory studies on time and required additional funding from the limited funds for dental research. In a survey from Australia, nearly 90% of the researchers identified delays in achieving project milestones, and 65% indicated delays in acquiring new funding [26].

### 3.4. Potential Challenges in Dental Students’ Hesitancy about the COVID-19 Vaccine

Because of aerosol-producing dental procedures, dental healthcare workers are at an increased risk of exposure to COVID-19. Vaccination of dental providers including dental students is critical for clinical-based training. However, the global acceptance level of dental students of COVID-19 vaccines was suboptimal [27]. In a global survey involving 6639 students from 22 countries, 22.5% of dental students were hesitant, and 13.9% rejected COVID-19 vaccines [27]. Another study in the United States showed that, although nearly all participants agreed they would likely be exposed to COVID-19, only 56% wanted to receive a COVID-19 vaccine, and 16.3% rejected COVID-19 vaccines even if mandated [28]. Recently, a meta-analysis revealed that vaccination acceptance of dental students was 60.5%, much lower than that of dental practitioners (81.1%) [29]. In addition, although dental students were more likely to be exposed to COVID-19, they were 2.7 times less likely than medical students to receive the vaccine [30]. Therefore, the attitude of dental students to the COVID-19 vaccine is a potential challenge for dental education.

### 3.5. Opportunities for Distance Education in Dental Education

Conventional teaching is based on the “face-to-face” approach, and in a typical classroom situation, a teacher often has to deal with several students at the same time. Distance education provides students with a flexible, self-contained, and accessible learning environment. Unsurprisingly, the most frequently reported advantage of distance education is flexible access. Other reported advantages included creativity in learning, enhanced critical thinking skills, increased student responsibility, etc. [31]. Interestingly, the private chat option allowed more anonymity in distance e-learning, and so shy students were more likely to ask questions, spearheading more discussion. On the other hand, the most frequently reported limitations of distance education included limited computer access, equipment, and skills of students and decreased student interaction and discussion [31]. In both dental education [31,32] and medical education [33], distance education has been shown to be as effective as traditional in-person education. Therefore, blended learning, which combines distance education and conventional teaching methods, was advocated even before the COVID-19 pandemic [34,35].

Due to lockdowns and guidelines during the COVID-19 pandemic, conventional teaching, which is limited by the classroom, was virtually suspended in the global world. Therefore, distance education became increasingly important. In a survey of 21 Chinese institutions, all institutions suspended conventional teaching and provided distance education during COVID-19, compared with 6/21 pre-COVID-19. The total number of online courses for 10 weeks was 33 pre-COVID-19, and that for two weeks during the epidemic was 119 [18]. During the pandemic, distance education was the prevailing action taken in different dental colleges [6,19]

Before the COVID-19 pandemic, distance education was found to be an effective tool to enhance the learning experience [36], considered a helpful adjunct and not a replacement for conventional educational methods [37]. Unsurprisingly, during the COVID-19 pandemic, students and instructors were satisfied with the rapid transition to distance education [38]. It has been also reported in a recent survey that almost 70% of dentists and hygienists attribute great importance to online courses [39].

In general, most students showed a positive attitude toward distance education (Table 1). For example, almost all students in the dental school of Justus-Liebig-University in Giessen found that distance education was a good substitute in the time of the COVID-19 pandemic, and both students and instructors wanted to maintain distance education in the future [40]. In a survey in Taipei including 473 students, 77% of them showed positive attitudes to distance education [41]. Students were very satisfied with the provision, quality, and benefit of distance digital education [42]. When compared with distance education, more students preferred conventional education (Table 1). For example, in the Dentistry Universitas Indonesia, although students agreed that distance education was a more efficient learning method (52.6%), it provided more time to study (87.9%) and to review study materials (87.3%), only 44.2% dental students preferred distance education over conventional education [43]. In Turkey, 61.9% of dental students preferred conventional education [44]. There was a big variation in the perceptions of distance education between these studies. Some of this can be attributed to the differences in internet connection and lecture system between those areas, which were the common difficulties that students experienced. Students usually prefer online lectures, instead of practical sessions or anatomy (Table 1). The studies might draw similar conclusions if they focused on a specific aspect of distance education.

In clinical dental education, problem-based learning (PBL) is widely used, due to the activation of learning motivation. During the suspension of clinical activities, both students and instructors preferred online case-based discussions as an alternative method of clinical dental education [6]. Visual, auditory, reading/writing, and kinaesthetic are predominant domains in clinical dental education. Distance PBL can facilitate visual/spatial, auditory, and reading/writing domains. However, distance PBL is not a reliable tool to facilitate kinaesthetic learning, because it does not allow hands-on practice. Clinical training is one of the big problems of distance education, therefore, interactive tools were strongly suggested by dental students [45] (Table 1).

### 3.6. Opportunities for Teledentistry in Dental Education

Teledentistry is a new term in the past decades, and its roots lie in telemedicine. The definition of telemedicine, according to the Association of American Medical Colleges is “Telemedicine is the use of telecommunications technology to send data, graphics, audio, and video images between participants who are physically separated (i.e., at a distance from one another) for the purpose of clinical care.” Teledentistry was found to reduce appointment waiting times and wasted clinical time for practitioners, the travel cost and time for patients [46]. Before the COVID-19 pandemic, a systematic review indicated that teledentistry is as reliable as face-to-face clinical observations in a range of settings, including the following; screening, orthognathic examination, orthodontic treatment, remote detection of root canals, indications for oral surgery, diagnosis of orofacial diseases, and management of endogenous infections [46]. However, it is difficult to diagnose the extent of demineralization accurately [47]. Overall, due to the efficiencies, a high level of acceptability of teledentistry has been reported [46]. However, teledentistry has not been widely accepted, because some barriers have limited its adoption, including reimbursement issues, license regulations, costs, limitations in physical examinations, and equipment required [48,49,50].

Due to the advances of COVID-19, the routines of dental offices were enormously affected and elective services were temporarily suspended, with only emergency and urgent procedures being performed. Teledentistry has become a boon for patients during the COVID-19 lockdown [51,52,53]. In addition, dentists benefited from teledentistry during the COVID-19 pandemic, because it allowed the monitoring of patients, reduction of costs and limitation of human contact, decreasing the risk of (COVID-19 dissemination [54]. Therefore, teledentistry became a viable alternative for more and more people [55,56,57]. The satisfaction level of patients using teledentistry was extremely high during the COVID-19 pandemic [58]. However, a recent meta-analysis reported that dental practitioners had a high level of awareness (70.4%) and attitude (72.5%) towards teledentistry, but a moderate level of knowledge (57.9%) and poor practice level (35.8%) during the COVID-19 pandemic [59]. These interesting results indicated that dental practitioners agreed that teledentistry is a brilliant invention, but they were unclear about the required knowledge and skills [59]. One probable explanation is that dental practitioners were not properly trained in teledentistry. Therefore, opportunities for teledentistry in dental health have come. However, facing the barriers of teledentistry, dental education should pay more attention to teledentistry. To increase the acceptance of teledentistry and benefit more people during the COVID-19 pandemic, dentists and dental students must be trained adequately and educated about this technology.

### 3.7. Opportunities for Dental Emergency Education

Healthcare emergencies are considered life-threatening conditions. Urgent conditions are not life-threatening; however, failure to address urgent conditions in a timely manner may lead to an emergency. Therefore, a dental emergency is defined as a potentially life-threatening condition. During the COVID-19 pandemic, since most of the dental hospitals and dental clinics were closed, in most countries only emergency units were open for patients to treat uncontrolled bleeding, cellulitis, or a diffuse soft tissue bacterial infection with intra-oral or extra-oral swelling that potentially compromises the patient’s airway, or trauma involving facial bones [16]. Unquestionably, a dental emergency is an important part of dental healthcare and played a key role in the COVID-19 pandemic. In addition, a dental emergency is a suitable field for students to learn about acute dental needs and the management of pain and anxiety [60]. However, dental emergency education has been neglected for a long time. No dental educator would dispute the need for a systematic focus on the approach to urgent care in the dental school setting, but very little has been written on the subject. A survey focusing on dental emergency education in the U.S. revealed that dental emergency education is likely the most variable component of the dental curriculum [61]. Since COVID-19 has brought dental emergency into the core area of dental healthcare, it is time to guide the development of a more formalized curriculum for this area of care.

## 4. Discussion

Despite the global recovery, dental education will continue to face the challenges outlined above. While some students have returned to campus, the number of students per clinical session is strictly limited in order to maintain adequate social distancing, and online courses remain an important part of education. There are limitations to clinical training as well. It is noteworthy that the loss of non-clinical teaching and clinical-based training caused by the COVID-19 pandemic has generated high levels of negative mood in students, and may have resulted in them lacking confidence when faced with treating patients. Much work is required in dental education to repair the specialized knowledge, skills, and psychological health.

For psychological health, there were various coping mechanisms deployed by dental students, including watching television, reading, spending time on social media, and spending time with family [62]. In addition, video games, limiting the amount of alcohol or not drinking alcohol at all, and helping others are good for psychological health, according to the World Health Organization. Regular physical activity can also reduce the risk of depression and improve overall feelings. Considering students’ positive attitude to distance education, schools could try to improve their online courses to pass on specialized knowledge, such as providing training in using the system and interactive lectures (Table 1). Facing the challenge of specialized skills, students could join in a dental emergency where they have a chance to learn the management of dental pain and anxiety and basic specialized skills. Teledentistry also provides extensive opportunities for the clinical capability of dental students [46]. In addition, clinical-based training can benefit from distance education with excellent kinaesthetic learning. For dental research, Divesh et al. suggested some practical measures, such as balancing access to the laboratory with COVID-19 preventive regulations and flexibility in the administration of research grants [25]. To reduce hesitancy towards the COVID-19 vaccine, educational curricula about its safety and effectiveness, to promote the uptake of the COVID-19 vaccine, are required. Moreover, high-quality videos about COVID-19 and vaccines should be disseminated by health organizations, because 27.5% of the most popular videos about COVID-19 on YouTube contained misleading information [63].

Considering the far-reaching impact of the pandemic on society, dental educators should consider reforming the traditional dental education in some respects, to suit dental health under and post-COVID-19 pandemic. The COVID-19 pandemic has magnified the advantages of teledentistry and dental emergency. In the near future, these will continue to play important roles in dental healthcare. However, the practice of teledentistry is not as good as the awareness of teledentistry, owing to lack of training. Hence, a more formalized curriculum and adequate training for teledentistry will prepare dental students for future work. The COVID-19 pandemic also led to an increase in distance education. Although, compared with distance education, most students preferred conventional education [43,44], distance education had some irreplaceable advantages during the pandemic. Considering that clinical-based training relies highly on hands-on practice, we need enhanced visual, auditory and kinaesthetic distance education. Three-dimensional modeling and animations have been shown to increase students’ spatial visualization [64] and positive perception of education [65]. Augmented reality (AR)/virtual reality (VR)-based simulation devices were exclusively used as adjunct instruction tools during the pre-clinical training period [66,67,68]. In the entertainment industry, three-dimensional modeling and animations have brought photorealistic virtual worlds to life with a kinesthetic sense distantly. Therefore, distance dental education can also benefit from technology to enhance visual, auditory, and kinaesthetic learning.

## 5. Conclusions

COVID-19 not only brought physical and psychological health problems to dental students, but also raised challenges to the course of dental education, including non-clinical teaching, clinical-based training, and students’ research. In addition, hesitancy about the COVID-19 vaccine could increase those challenges. On the other hand, the COVID-19 pandemic has increased the demand and brought awareness of the advantages of distance education, teledentistry, and dental emergency. Considering the slow global recovery, we believe they will continue to play important roles in dental healthcare and education.

## Figures and Tables

**Table 1:** Dental students’ perceptions towards distance education.

Author	Region	Positive Attitude to Distance Education	Prefer Distance Education over Conventional Education	Issues or Recommendations
Hattar, S. et al.	Jordan	66%	67% preferred distance lectures over conventional lectures	Issues with clinical training (87%)
Schlenz, M.A. et al.	Germany	Almost all	63.20%	Not mentioned
Singal, A. et al.	India	50%	88% thought live dissections helped them to understand dental anatomy	Issues with proper hardware, internet connection (83%) and time management, distracted by home comforts or discomforts, lack of self-motivation(68%)
Cheng, H.C. et al.	Taiwan, China	77%	Not mentioned	Not mentioned
Goob, J. et al.	Germany	100%	82.5% preferred distance lectures over conventional lectures	Not mentioned
Amir, L.R. et al.	Indonesia	37.7% positive, 23.9% neutral	25.0% preferred distance education, 13% neutral	Issues with technological conditions (22.6%) and motivation (45.3%). Recommend interactive lectures, limiting the lectures to 30 min, and training on the system.
Sarialioglu Gungor, A. et al.	Turkey	Not mentioned	44.20%	Issues with the internet connection, the extra financial burden for the internet quota, and difficulty focusing while learning online.
Hassan, R. et al.	Egypt	73.5% with online lectures, 29.7% with practical sessions	Not mentioned	Recommend training on e-learning tools and computer skills, enhancing the interaction between students and teachers, 10–15 min at the end of each lecture for students’ questions, weekly online assessment, and practical learning through interactive tools.
